# Supplementary material for: A TRIM21-based bioPROTAC highlights the therapeutic benefit of HuR degradation
Source: Nat Commun. 2023 Nov 4;14:7093. doi: 10.1038/s41467-023-42546-2 (PMC10625600; doi:10.1038/s41467-023-42546-2)
Supplement: Supplementary file 1 — Supplementary Information [file 41467_2023_42546_MOESM1_ESM.pdf]

**Supplementary Information**

# Supplementary Figure 1

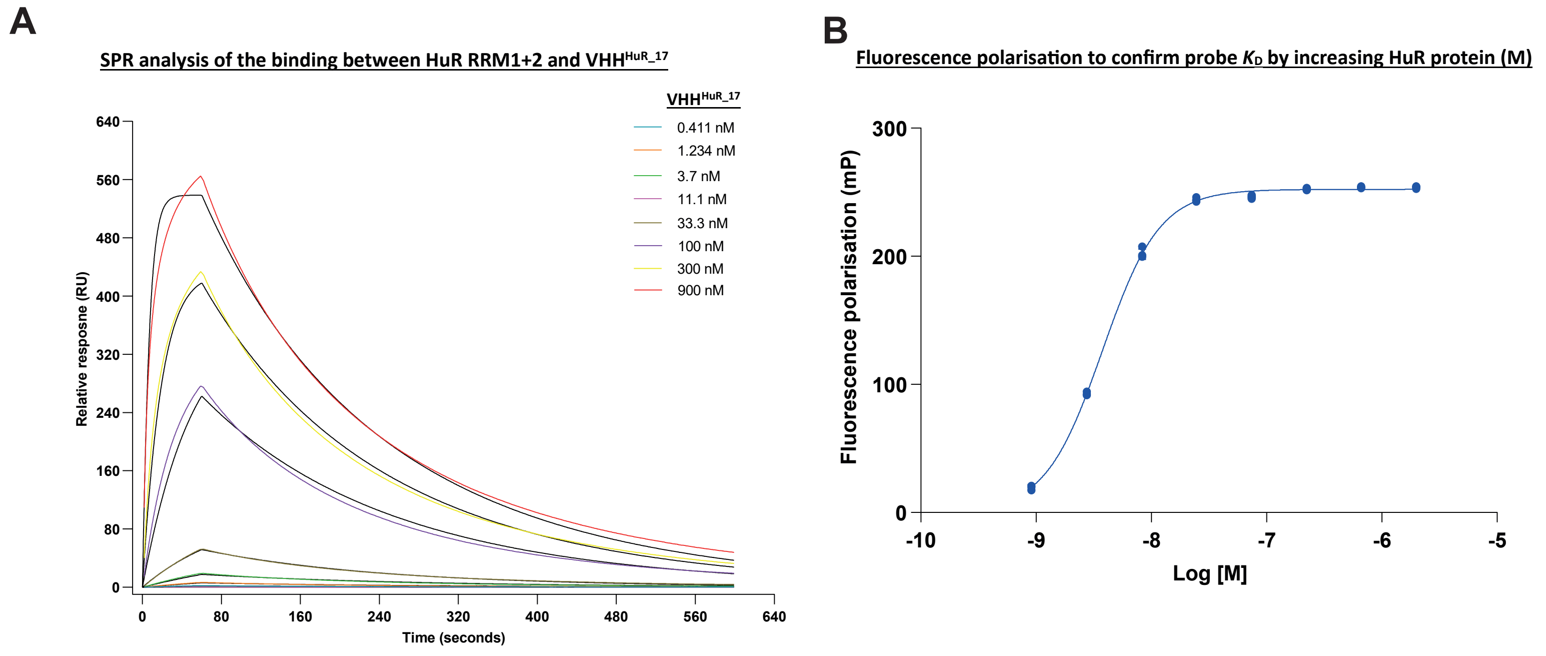


**Supplementary Figure 1. Characterisation of a lead VHH$^{HuR}$ which binds and inhibits HuR.** A) Representative example of SPR analysis showing the binding between HuR RRM1+2 and VHH$^{HuR_17}$ at a range of concentrations. B) Graph of fluorescence polarisation to confirm probe $K_D$ by increasing HuR protein (x-axis) when incubated with 10 nM Msi1-FITC labelled RNA probe (n=1; four technical repeats per concentration, line = non-linear curve fit). Source data are provided as a Source Data file.

# Supplementary Figure 2

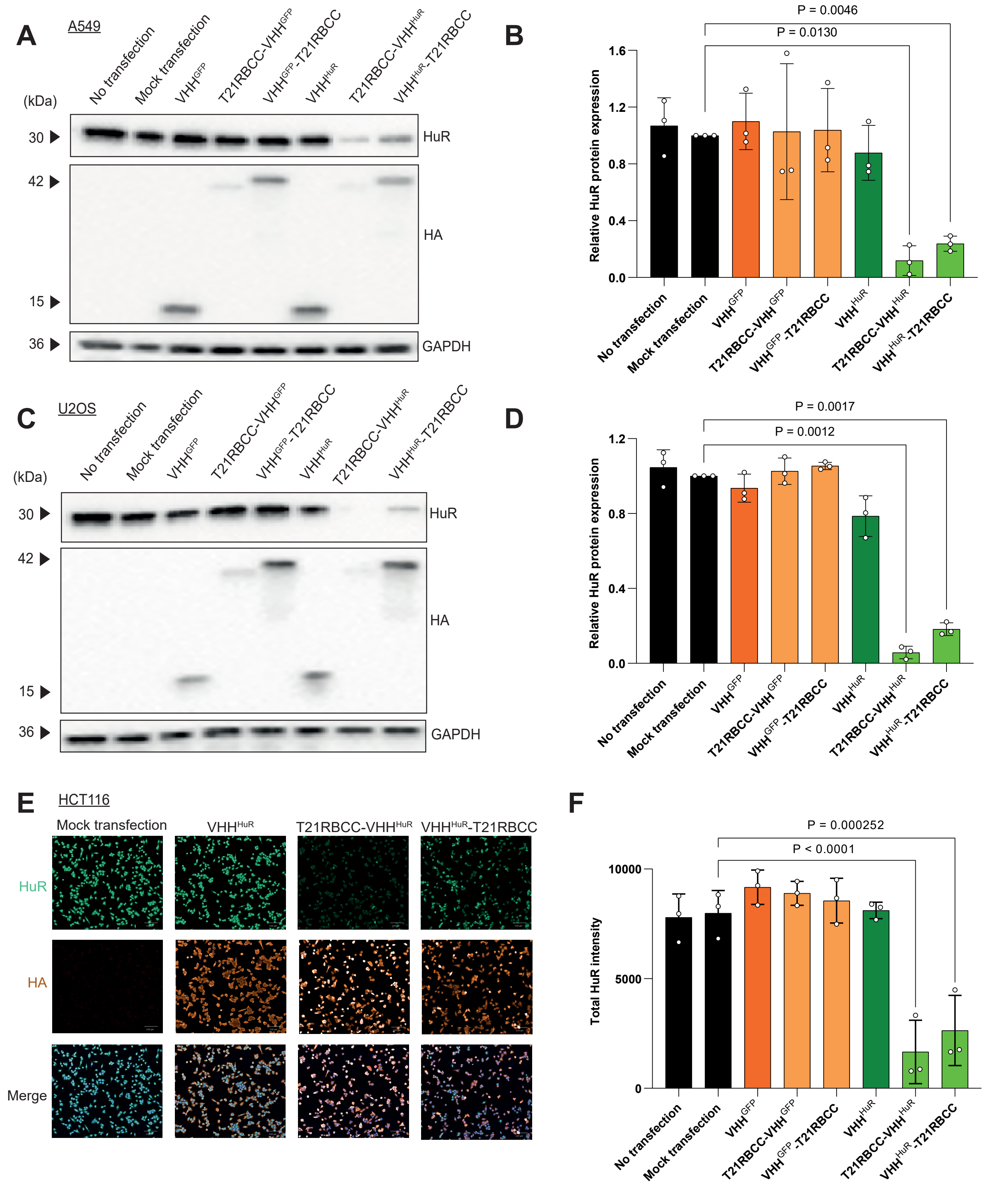


**Supplementary Figure 2. T21RBCC-VHH$^{HuR}$ bioPROTACs degrade HuR in multiple cell lines.** Representative immunoblot and associated densitometry following mRNA transfection of HA-tagged bioPROTAC constructs in the A549 (A-B) or U2OS (C-D) cell lines for 18 hours. (E-F) Representative images and quantification of immunofluorescence of total HuR intensity in HCT116 cells 18 hours post-transfection with HA-tagged bioPROTAC constructs followed by staining with HuR (green), HA (red) and DAPI (blue) (n=3). Blots shown in (A) and (C) are representative from n=3 biologically independent samples per group. Data in (B), (D) and (F) show the mean and standard deviation from n=3 biologically independent samples per group. Statistical significance was calculated using a one-way ANOVA and post-hoc test. Source data are provided as a Source Data file.

## Supplementary Figure 3

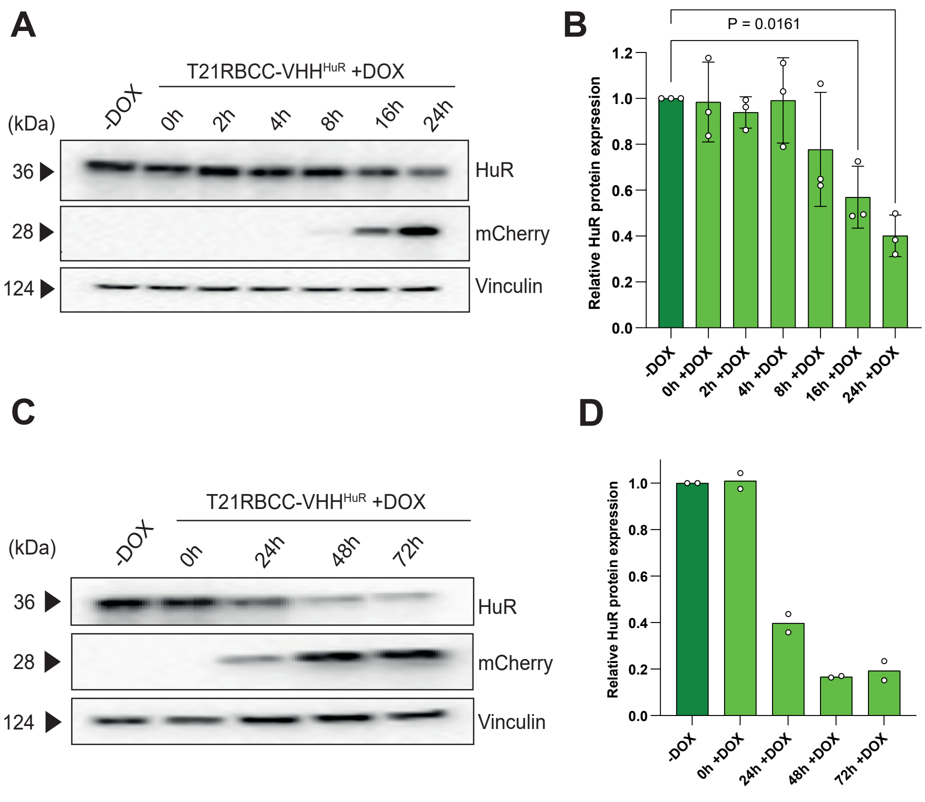


**Supplementary Figure 3. BioPROTAC expression and HuR degradation in the T21RBCC-VHH$^{HuR}$ ODIn cell line.** Representative immunoblot and associated densitometry following doxycycline induction of the HCT116-ODIn T21RBCC-VHH$^{HuR}$ cell line between 0-24 hours (A-B) or 0-72 hours (C-D) post-doxycycline induction. Blots shown in (A) and (C) are representative from n=3 (A) or n=2 (C) biologically independent samples per group. Data in (B) shows the mean and standard deviation, and in (D) mean alone, for densitometry measurements. Statistical significance was calculated using a one-way ANOVA and post-hoc test. Source data are provided as a Source Data file.

# Supplementary Figure 4

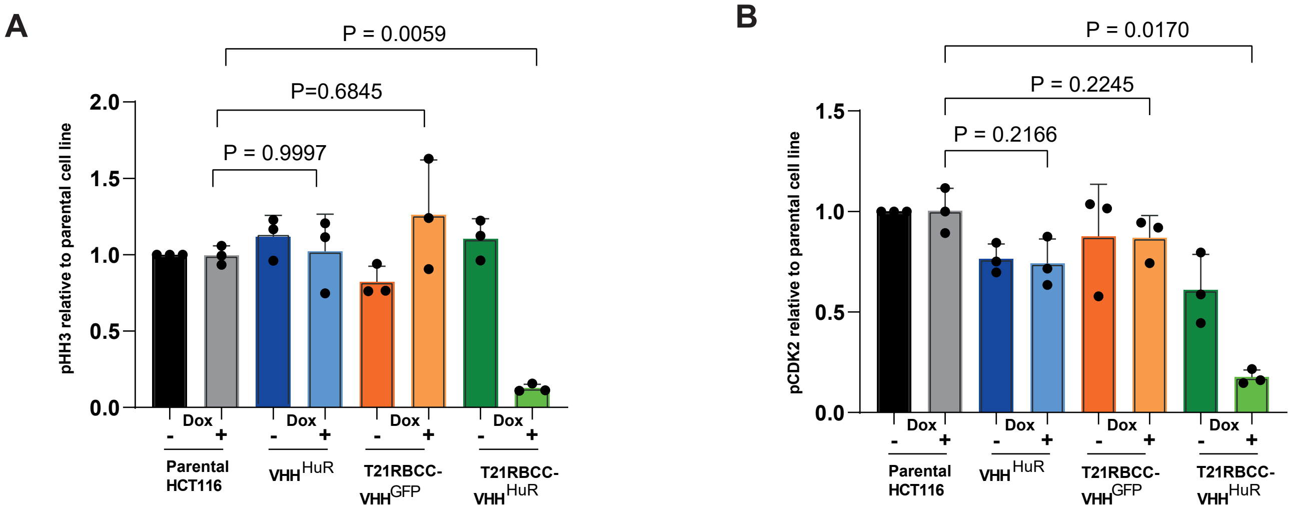


**Supplementary Figure 4. BioPROTAC expression and HuR degradation leads to a reduction in cell cycle markers pHH3 and pCDK2 in ODIn cell lines.** Densitometry, (A) pHH3 and (B) pCDK2, following doxycycline induction of the HCT116-ODIn T21RBCC-VHH$^{HuR}$ cell line (dark and light green) and related controls (parental cells, black and grey; VHH$^{HUR}$ alone cells, dark and light blue; T21RBCC-VHH$^{GFP}$ cells dark and light orange). Data in (A) and (B) show the mean and standard deviation from n=3 replicates of immunoblot of samples described in Fig. 2A and 2B. Statistical significance was calculated using a one-way ANOVA and post-hoc test. Source data are provided as a Source Data file.

# Supplementary Figure 5

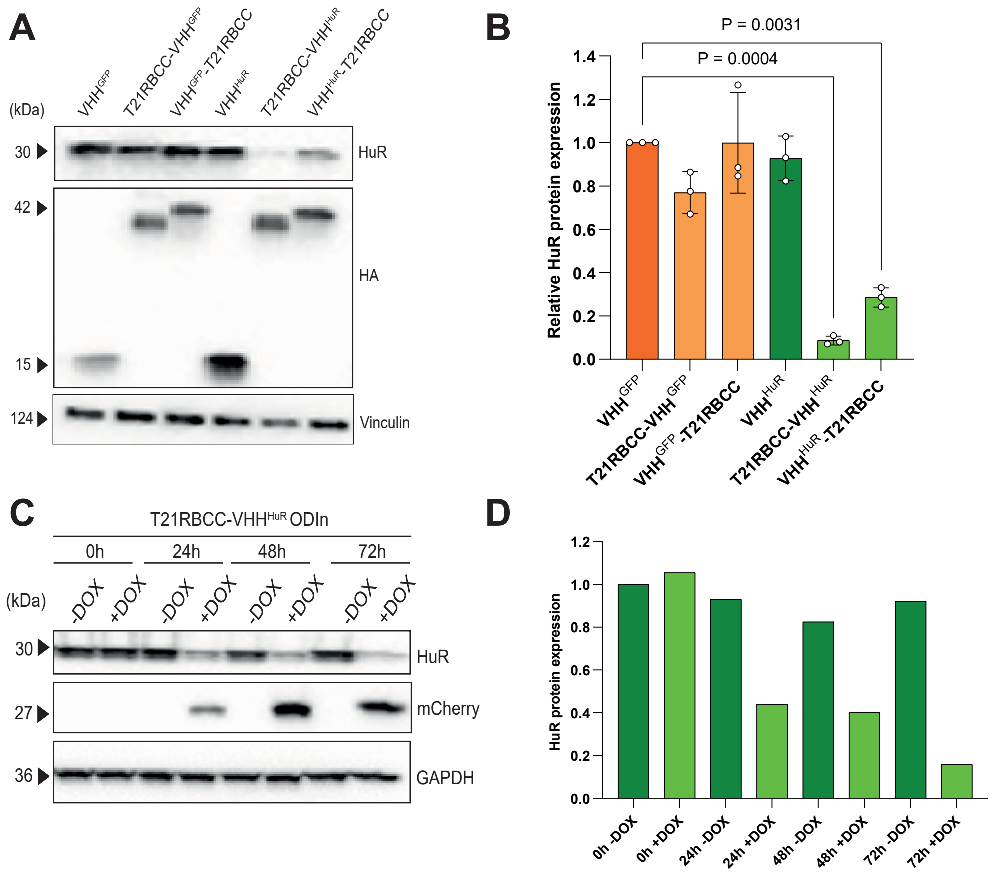


**Supplementary Figure 5. Confirmation of HuR degradation in samples prepared for mass spectrometry.** (A-B) Representative immunoblot and associated densitometry following mRNA transfection of bioPROTAC constructs in the HCT116 cell line for 18 hours. Blot shown in (A) is representative from n=3 biologically independent samples per group. Data in (B) shows the mean and standard deviation from n=3 biologically independent samples per group. Statistical significance was calculated using a one-way ANOVA and post-hoc test. (C-D) Immunoblot and associated densitometry of the HCT116-ODIn T21RBCC-VHH$^{HuR}$ cell line at 0-72 hours post-doxycycline (n=1). Source data are provided as a Source Data file.

# Supplementary Figure 6

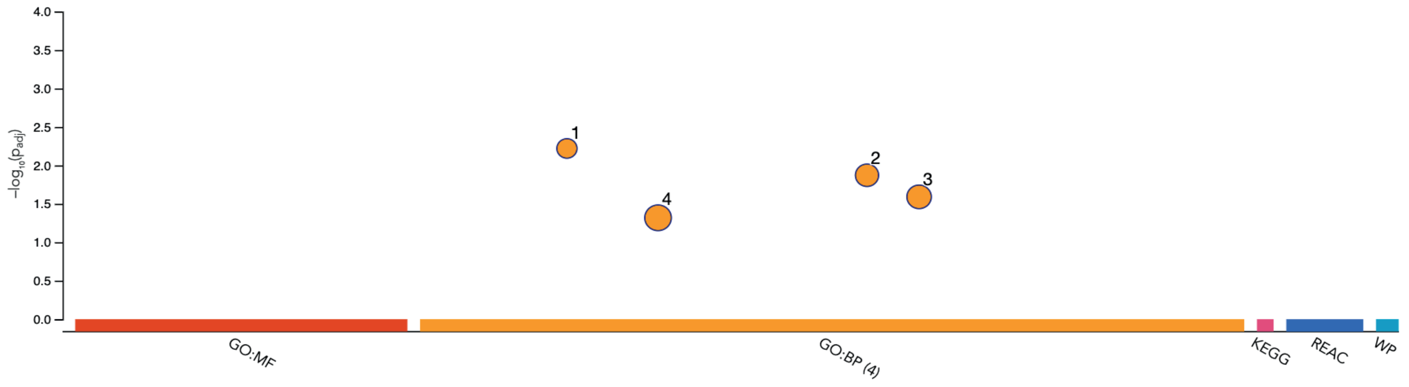


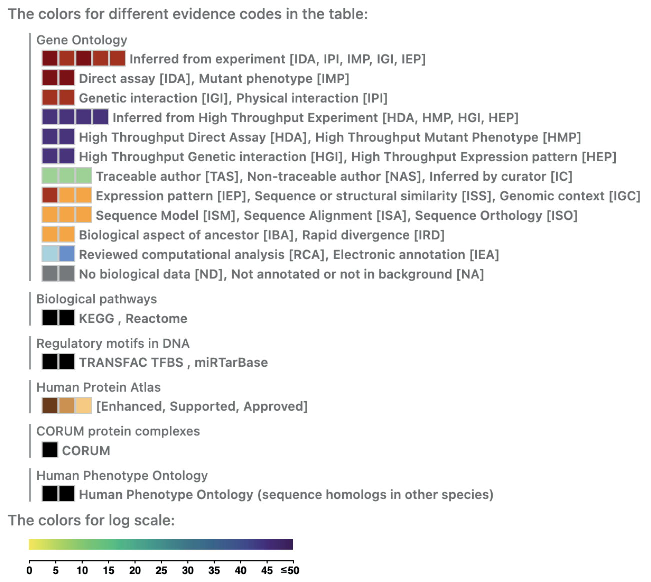


| ID | Source | Term ID | Term Name | $p_{adj}$ (query_1) |
|---|---|---|---|---|
| 1 | GO:BP | GO:0015711 | organic anion transport | $6.018\times10^{-3}$ |
| 2 | GO:BP | GO:0055085 | transmembrane transport | $1.343\times10^{-2}$ |
| 3 | GO:BP | GO:0065008 | regulation of biological quality | $2.563\times10^{-2}$ |
| 4 | GO:BP | GO:0032501 | multicellular organismal process | $4.802\times10^{-2}$ |

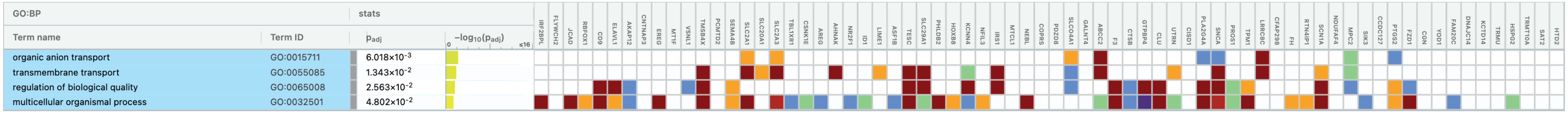


**Supplementary Figure 6. GO, KEGG, Reactome and WikiPathways enrichment analysis.** Analysis for the 71 significantly (Fisher's one-sided test) down- and up-regulated proteins at both 48-72 hours due to T21RBCC-VHH$^{HuR}$ expression using g:Profiler (https://biit.cs.ut.ee/gprofiler/gost) (Raudvere, U., et al., 2019).

# Supplementary Figure 7

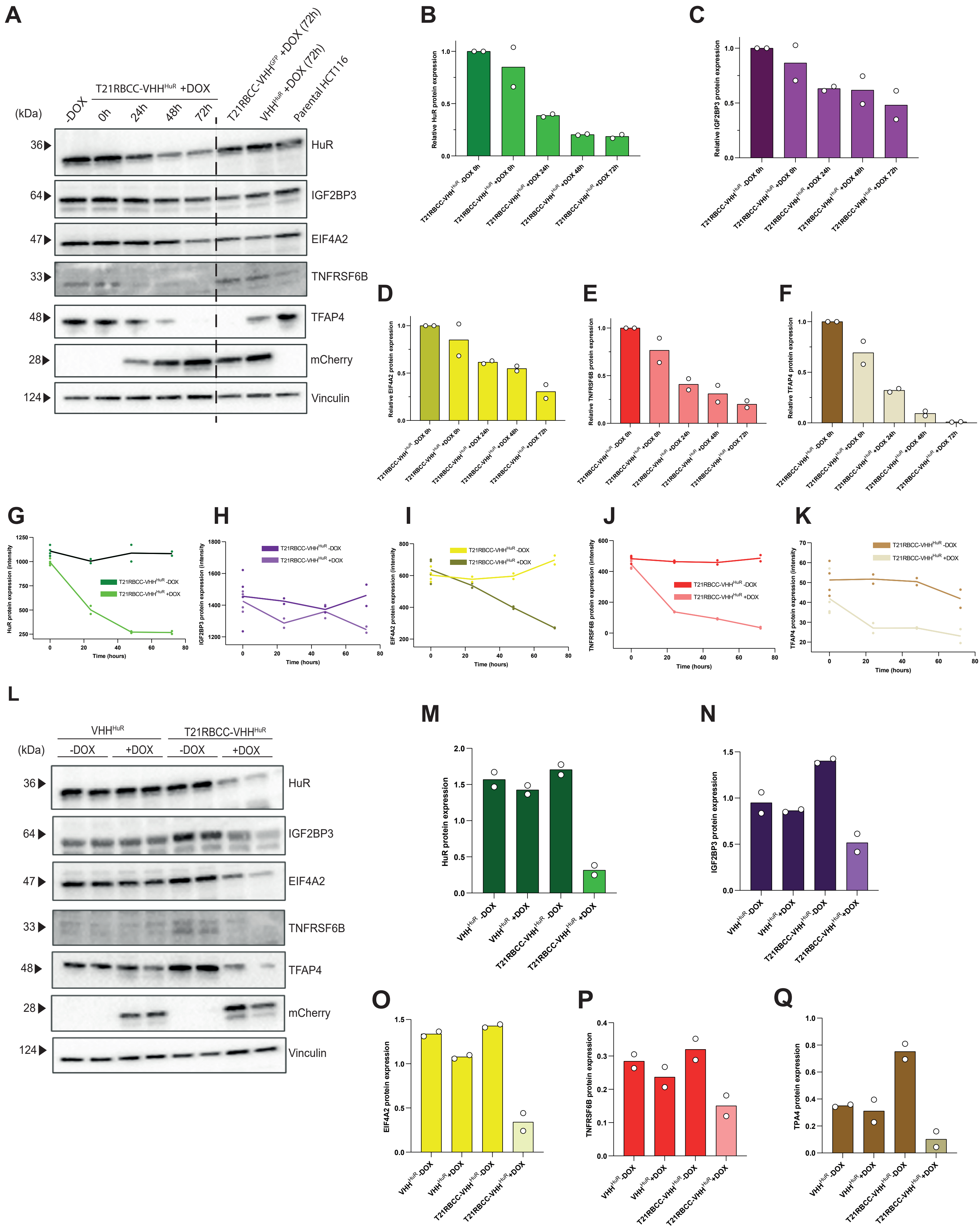

**Supplementary Figure 7. T21RBCC-VHH$_{HuR}$ decreases the expression of proteins of interest.** Representative immunoblots and associated densitometry (A-F) or protein expression intensity from mass spectrometry-based measurements (G-K) for HuR, IGF2BP3, EIF4A2, TNFRSF6B and TFAP4 in the HCT116-ODIn T21RBCC-VHH$_{HuR}$ cell line at 0-72 hours post-induction. Representative immunoblots and associated densitometry for HuR, IGF2BP3, EIF4A2, TNFRSF6B and TFAP4 in the T21RBCC-VHH$_{HuR}$ from mouse xenograft tumours (L-Q). Blots shown in (A) and (L) are representative from n=2 biologically independent samples per group. Data in (B-F) and (M-Q) show the mean and individual data points from n=2 biologically independent samples per group. Mass spectrometry data (G-K) shows technical repeats for each timepoint from one biologically independent sample with line showing mean value. Source data are provided as a Source Data file.

# Supplementary Figure 8

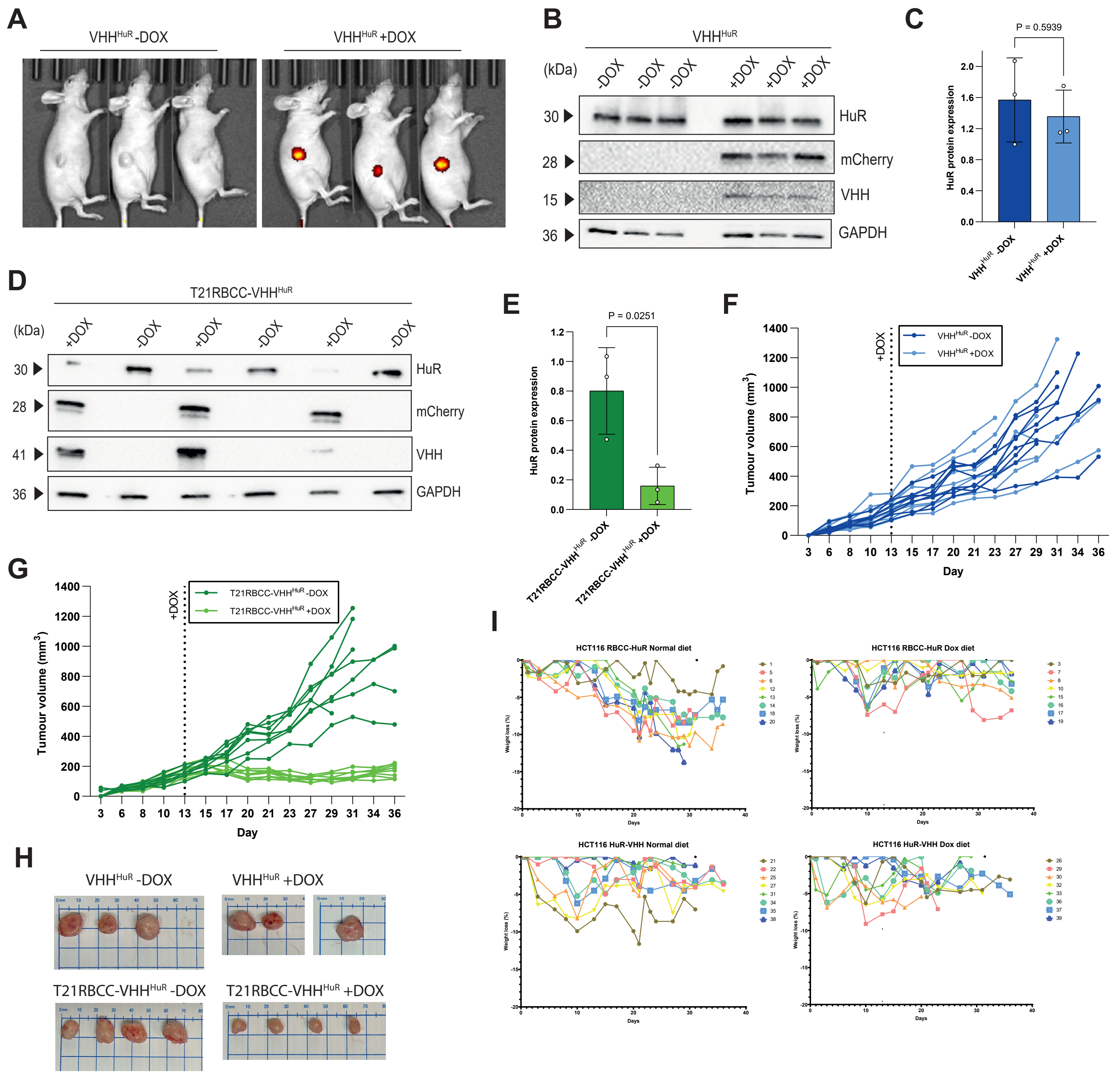


**Supplementary Figure 8. Validation and evaluation of mouse xenograft models - T21RBCC-VHH$^{HuR}$ arrests tumour growth *in vivo*.** (A) Confirmation of mCherry fluorescence in mice with VHH$^{HuR}$ xenografts receiving a standard or doxycycline-containing diet. Representative immunoblot and associated densitometry of lysates from VHH$^{HuR}$ (B-C) or T21RBCC-VHH$^{HuR}$ (D-E) xenografts in mice receiving a standard or doxycycline-containing diet. Line graph of tumour volumes (mm$^3$) from day 3-36 of VHH$^{HuR}$ (F) or T21RBCC-VHH$^{HuR}$ (G) xenografts in mice receiving a standard or doxycycline-containing diet (n=8 – data for individual mice displayed). (H) Representative images of tumours resected from VHH$^{HuR}$ or T21RBCC-VHH$^{HuR}$ xenografts. (I) Graphs of individual mouse weight for each group for the duration of the *in vivo* study. Blots shown in (B) and (D) are representative from n=3 biologically independent samples per group. Data in (C) and (E) show the mean and standard deviation from n=3 biologically independent samples per group. Statistical significance was calculated using an unpaired two-tailed t-test. Source data are provided as a Source Data file.
