# Supplementary material for: A TRIM21-based bioPROTAC highlights the therapeutic benefit of HuR degradation
Source: Nat Commun. 2023 Nov 4;14:7093. doi: 10.1038/s41467-023-42546-2 (PMC10625600; doi:10.1038/s41467-023-42546-2)
Supplement: Supplementary file 3 — Reporting Summary [file 41467_2023_42546_MOESM3_ESM.pdf]

nature portfolio

Corresponding author(s): James Hunt; Alice Fletcher

Last updated by author(s): Oct 30, 2023

Reporting Summary

Nature Portfolio wishes to improve the reproducibility of the work that we publish. This form provides structure for consistency and transparency in reporting. For further information on Nature Portfolio policies, see our Editorial Policies and the Editorial Policy Checklist.

Statistics

For all statistical analyses, confirm that the following items are present in the figure legend, table legend, main text, or Methods section.

n/a	Confirmed	
☐	☒	The exact sample size (*n*) for each experimental group/condition, given as a discrete number and unit of measurement
☐	☒	A statement on whether measurements were taken from distinct samples or whether the same sample was measured repeatedly
☐	☒	The statistical test(s) used AND whether they are one- or two-sided<br>*Only common tests should be described solely by name; describe more complex techniques in the Methods section.*
☒	☐	A description of all covariates tested
☐	☒	A description of any assumptions or corrections, such as tests of normality and adjustment for multiple comparisons
☐	☒	A full description of the statistical parameters including central tendency (e.g. means) or other basic estimates (e.g. regression coefficient) AND variation (e.g. standard deviation) or associated estimates of uncertainty (e.g. confidence intervals)
☐	☒	For null hypothesis testing, the test statistic (e.g. *F*, *t*, *r*) with confidence intervals, effect sizes, degrees of freedom and *P* value noted<br>*Give P values as exact values whenever suitable.*
☒	☐	For Bayesian analysis, information on the choice of priors and Markov chain Monte Carlo settings
☒	☐	For hierarchical and complex designs, identification of the appropriate level for tests and full reporting of outcomes
☒	☐	Estimates of effect sizes (e.g. Cohen's *d*, Pearson's *r*), indicating how they were calculated

Our web collection on statistics for biologists contains articles on many of the points above.

Software and code

Policy information about availability of computer code

Data collection

For immunoblot detection we used: Imagelab v6.l.0.07.
For colony formation detection we used: GelCount vl.1.2.0.
For proteomic data collection we used: Spectronaut SN14.10.201222 (with Pulsar search engine), Biognosys; Perseus vl.6.15.0; R v3.6.0; Python v3.8.5; Reference proteome (to analyse proteomic data) UniProt human proteome database [https://www.uniprot.org/proteomes/UP000005640] UP000005640, 96,797 entries.
For SPR we used: Biacore Control Software 3.2.1

Data analysis

For immunoblot analyses we used: Imagelab v6.l.0.07 TO ADD JON'S
For colony formation analyses we used: GelCount vl.1.2.0
For SPR we used: Biacore Evaluation Software 3.2.1
For immunofluorescence analyses we used: Columbus v2.9.l.532
For statistical analyses we used: Graph Pad Prism v9.0.0
For proteomic analyses we used: R v3.6.0; Python v3.8.5; Spectronaut (with Pulsar search engine (Version SN14.10.201222));
Perseus vl.6.15.0;
UniProt human proteome database [https://www.uniprot.org/proteomes/UP000005640];
UniProtKB (online version) UniProtKB keywords linked to biological processes https://www.uniprot.org/;
BioGRID 4.4 (online version) BioGRID https://thebiogrid.org/ (Oughtred, R., et al.,2020);
limma v3.42.2 R Bioconductor https://bioconductor.org/packages/release/bioc/html/limma.html;
tidyverse vl.3.0 The Comprehensive R Archive Network https://cran.r-project.org/web/packages/tidyverse/index.html;

data.table vl.13.2 The Comprehensive R Archive Network https://cran.r-project.org/web/packages/data.table/index.html;
Venn Diagram vl.6.20 The Comprehensive R Archive Network https://cran.rproject.org/web/packages/VennDiagram/index.html; ggplot2 v3.3.2 The Comprehensive R Archive Network https://cran.r-project.org/web/packages/ggplot2/index.html;
ggrepel v0.9.1 The Comprehensive R Archive Network https://cran.r-project.org/web/packages/ggrepel/index.html;
g:Profiler (https://biit.cs.ut.ee/gprofiler/gost).
Code used for proteomic analyses is deposited on GitHub at https://github.com/AstraZeneca/trim21-bioprotac.

For manuscripts utilizing custom algorithms or software that are central to the research but not yet described in published literature, software must be made available to editors and reviewers. We strongly encourage code deposition in a community repository (e.g. GitHub). See the Nature Portfolio guidelines for submitting code & software for further information.

Data

Policy information about availability of data

All manuscripts must include a data availability statement. This statement should provide the following information, where applicable:

- Accession codes, unique identifiers, or web links for publicly available datasets
- A description of any restrictions on data availability
- For clinical datasets or third party data, please ensure that the statement adheres to our policy

Data supporting the findings of this study are presented within the article and supplemental information. Source data are provided within this manuscript, and the mass spectrometry proteomics data have been deposited to the ProteomeXchange Consortium via the PRIDE (Perez-Riverol, Y., et al., 2022) partner repository with the dataset identifiers PXD033221 (non-kinetic data; https://www.ebi.ac.uk/pride/archive/projects/PXDO33221) and PXD033222 (kinetic data; https://www.ebi.ac.uk/pride/archive/projects/PXDO33222). The human reference, GO, KEGG, Reactome and WikiPathways data sources that were used for the enrichment analyses are as follows: hsapiens (Human) - version: GRCh38.p13; GO – annotations BioMart release 2023-03-06; KEGG – KEGG FTP release 2023-03-27; Reaactome – annotations: BioMart release 2023-3-29; WikiPathways release 20230310. Python scripts were used to process the output obtained from Uniproed and barplots were generated using R and are available at the following web link (https://github.com/AstraZeneca/trim21-bioprotac).

Research involving human participants, their data, or biological material

Policy information about studies with human participants or human data. See also policy information about sex, gender (identity/presentation), and sexual orientation and race, ethnicity and racism.

Reporting on sex and gender	*Use the terms sex (biological attribute) and gender (shaped by social and cultural circumstances) carefully in order to avoid confusing both terms. Indicate if findings apply to only one sex or gender; describe whether sex and gender were considered in study design; whether sex and/or gender was determined based on self-reporting or assigned and methods used.*<br>*Provide in the source data disaggregated sex and gender data, where this information has been collected, and if consent has been obtained for sharing of individual-level data; provide overall numbers in this Reporting Summary. Please state if this information has not been collected.*<br>*Report sex- and gender-based analyses where performed, justify reasons for lack of sex- and gender-based analysis.*
Reporting on race, ethnicity, or other socially relevant groupings	*Please specify the socially constructed or socially relevant categorization variable(s) used in your manuscript and explain why they were used. Please note that such variables should not be used as proxies for other socially constructed/relevant variables (for example, race or ethnicity should not be used as a proxy for socioeconomic status).*<br>*Provide clear definitions of the relevant terms used, how they were provided (by the participants/respondents, the researchers, or third parties), and the method(s) used to classify people into the different categories (e.g. self-report, census or administrative data, social media data, etc.)*<br>*Please provide details about how you controlled for confounding variables in your analyses.*
Population characteristics	*Describe the covariate-relevant population characteristics of the human research participants (e.g. age, genotypic information, past and current diagnosis and treatment categories). If you filled out the behavioural & social sciences study design questions and have nothing to add here, write "See above."*
Recruitment	*Describe how participants were recruited. Outline any potential self-selection bias or other biases that may be present and how these are likely to impact results.*
Ethics oversight	*Identify the organization(s) that approved the study protocol.*

Note that full information on the approval of the study protocol must also be provided in the manuscript.

Field-specific reporting

Please select the one below that is the best fit for your research. If you are not sure, read the appropriate sections before making your selection.

☒ Life sciences ☐ Behavioural & social sciences ☐ Ecological, evolutionary & environmental sciences

For a reference copy of the document with all sections, see nature.com/documents/nr-reporting-summary-flat.pdf

Life sciences study design

All studies must disclose on these points even when the disclosure is negative.

Sample size	Power calculations using Russ Lenth's power tool (cited in the manuscript) were used to inform in vivo group sizes. For in vitro experiments, no power calculations were undertaken, with sample size taken as the number of repeats of each experiment.
Data exclusions	Four data points were excluded as an anomaly in Figure 1B - this has been highlighted in the source data file. No other data has been excluded.
Replication	Sample size is reported in Figure legends. All attempts at replication were successful. Technical repeats are individual samples prepared and measured on the same day, as part of the same experiment. All figures report n=2, or greater, independent biological repeats except:<br>Figure 1: (A) n=1; representative of four repeats<br>Figure 1: (B) n=1; three technical replicates plotted per A:D ratio; representative of three repeats<br>Figure 1: (D) n=1; four technical replicates plotted per concentration; representative of two repeats<br>Figure 1: (E) n=1; orthogonal confirmation of probe displacement described in Figure 1(D)<br>Figure 1: (F) n=1; repeated independently in HCT116 cells<br>Figure 3: (H-K) n=1; four time points captured (0h, 24h, 48h, 72h); HuR degradation confirmed by western blot (Supplementary Figure 5C-D) observations also independently confirmed by western blot (Supplementary Figure 7A-F; n=2)<br>Supplementary Figure 1A: n=1; representative of three independent SPR experiments using RRM1, 1+2, Full length (see Figure 1D)<br>Supplementary Figure 1B: n=1; four technical repeats plotted per concentration; verification of probe used in Figure 1 (D); representative of two repeats<br>Supplementary Figure 5C-D n=1; confirmation of HuR degradation in samples for proteomic time course (Figure 3 (H-K))
Randomization	Tumour-bearing mice engrafted with HCT116-ODln T21RBCC-VHHHuR or VHHHuR cell lines were size-matched and randomly assigned into experimental groups upon reaching an average tumour size of 150mm3 prior to receiving the doxycycline diet. For in vitro experiments samples were not randomised.
Blinding	Investigators were blinded when analysing tumour samples. For all other experiments, investigators were not blinded because experiments were performed by individual researchers. Experimental data are precise measurements and are not subjective.

Reporting for specific materials, systems and methods

We require information from authors about some types of materials, experimental systems and methods used in many studies. Here, indicate whether each material, system or method listed is relevant to your study. If you are not sure if a list item applies to your research, read the appropriate section before selecting a response.

Materials & experimental systems

n/a	Involved in the study	
☐	☒	Antibodies
☐	☒	Eukaryotic cell lines
☒	☐	Palaeontology and archaeology
☐	☒	Animals and other organisms
☒	☐	Clinical data
☒	☐	Dual use research of concern
☒	☐	Plants

Methods

n/a	Involved in the study	
☒	☐	ChIP-seq
☒	☐	Flow cytometry
☒	☐	MRI-based neuroimaging

Antibodies

Antibodies used	Mouse monoclonal anti-HuR [3A2] ThermoFisher Scientific #39-0600;<br>Rabbit monoclonal anti-HuR [D9W7E] Cell Signaling Technology #12582;<br>Rabbit anti-COXIV LI-COR #926-42214;<br>Mouse anti-TRIM21 [D-12] Santa-Cruz Biotechnology #sc-25351;<br>Goat anti-IgG (Fc) BioRad #5211-8004;<br>Rabbit polyclonal mCherryThermoFisher Scientific #PA5-34974;<br>Rabbit monoclonal anti-HA [C29F4] Cell Signaling Technology #3724;<br>Rabbit monoclonal anti-Camelid VHH Cocktail GenScript #A02014-200;<br>Rabbit monoclonal anti-GAPDH [Dl6Hll] Cell Signaling Technology #5174;<br>Rabbit monoclonal anti-Vinculin [E1E9V] Cell Signaling Technology #13901;<br>Mouse monoclonal anti-FLAG® M2 Sigma #F3165;<br>Rabbit polyclonal anti-IGF2BP3 ProteinTech #14642-1-AP;<br>Rabbit polyclonal anti-EIF4A2 Abcam #ab31218;<br>Rabbit polyclonal anti-TNFRSF6B (DcR3) Abcam #ab8405;

	Rabbit Polyclonal anti-TFAP4 Novus Biologicals (NBP1-89060)<br>Goat anti-mouse (HRP-conjugated) Cell Signaling Technology #7076;<br>Goat anti-rabbit (HRP-conjugated) Cell Signaling Technology #7074;<br>Rabbit anti-mouse (HRP-conjugated) Dako #P0260;<br>Goat anti-rabbit (HRP-conjugated) Invitrogen #31460;<br>Rabbit anti-goat (HRP-conjugated) Invitrogen #A27014;<br>Donkey anti-rabbit IgG, Alexa Fluor 568 ThermoFisher Scientific #A10042;<br>Goat anti-mouse IgG, Alexa Fluor 488 ThermoFisher Scientific #All00l;<br>pCDK/pHH3/Actin primary antibody cocktail, Abcam #ab136810;<br>IRDye 800CW Goat anti-Rabbit IgG secondary antibody, LI-COR, #926-32211;<br>Rabbit polyclonal anti-Myc HRP, Abcam, #ab1326
Validation	For each primary antibody used, the vendor has validated the quality via confirmation of target detection across a panel of cell lines/ tissues and/or has cited publications which have used the antibody. See:<br><br>https://antibodyregistry.org/AB_2533394<br>https://antibodyregistry.org/AB_2797964<br>https://antibodyregistry.org/AB_2783000<br>https://antibodyregistry.org/AB_617100<br>https://antibodyregistry.org/AB_2552323<br>https://antibodyregistry.org/AB_1549585<br>https://www.genscript.com/antibody/A02014-MonoRab_Rabbit_Anti_Camelid_VHH_Cocktail.html<br>https://antibodyregistry.org/AB_10622025<br>https://antibodyregistry.org/AB_2728768<br>https://antibodyregistry.org/AB_259529<br>https://antibodyregistry.org/AB_2122782<br>https://antibodyregistry.org/sAB_732123<br>https://www.abcam.com/products/primary-antibodies/dcr3-antibody-ab8405.html<br>https://www.novusbio.com/products/tfap4-antibody_nbp1-89060<br>https://antibodyregistry.org/AB_330924<br>https://antibodyregistry.org/AB_2099233<br>https://antibodyregistry.org/AB_2636929<br>https://antibodyregistry.org/sAB_228341<br>https://antibodyregistry.org/AB_2536079<br>https://antibodyregistry.org/AB_2534017<br>https://antibodyregistry.org/AB_2534069<br>https://www.abcam.com/products/panels/cell-cycle-pcdkphh3actin-wb-cocktail-ab136810.html<br>https://antibodyregistry.org/AB_621843<br>https://antibodyregistry.org/AB_877442<br>https://www.abcam.com/products/primary-antibodies/hrp-myc-tag-antibody-ab1326.html<br><br>For HuR, we additionally confirmed loss of HuR upon siRNA treatment with a HuR-targeting siRNA and observations for HuR, IGF2BP3, EIF4A2, TNFRSF6B and TFAP4 were all confirmed via proteomics.

Eukaryotic cell lines

Policy information about cell lines and Sex and Gender in Research

Cell line source(s)	HCT116 cells were obtained from the ECACC, and the A549, U2OS and RPE-1 cell lines were obtained from the ATCC.
Authentication	Cells were obtained from a commercial source. HCT116, A549 and U2OS were authenticated in-house via STR fingerprinting and hTERT-RPE-1 were by morphology.
Mycoplasma contamination	Cells were confirmed to be mycoplasma-free prior to use.
Commonly misidentified lines<br>(See ICLAC register)	No commonly misidentified cell lines were used.

Animals and other research organisms

Policy information about studies involving animals; ARRIVE guidelines recommended for reporting animal research, and Sex and Gender in Research

Laboratory animals	Athymic nude female mice were obtained at 8 weeks of age from Envigo. Mice were housed in specific pathogen-free and standardised environmental conditions according to UK Home Office regulations, on a 12/12 light/dark cycle, with ad libitum UV-treated water and sterilised RM1 rodent diet. Room temperature was held between 20-22 degrees and 50-60% humidity. Mice received irradiated aspen chip bedding, nesting material, a cardboard tunnel, and wooden chew blocks.

Wild animals	No wild animals were used in this study.
Reporting on sex	Only female mice were used in this study. Sex was not considered in the study design, as we concluded for a human xenograft tumour model the sex of the recipient would not have a significant impact on the results seen.
Field-collected samples	No field collected samples were used in this study.
Ethics oversight	These studies were conducted within the remit of a project licence approved by local Animal Welfare and Ethical Review Board (AWERB) committee and under a U.K. Home Office Project Licence in accordance with the U.K. Animals (Scientific Procedures) Act 1986 and EU Directive EU 2010/63/EU. Studies were performed according to the Home Office guidelines for the Care and Use of Laboratory Animals, and were also compliant with AstraZeneca policies on Bioethics and Good Statistical Practice in animal work.

Note that full information on the approval of the study protocol must also be provided in the manuscript.
